# New York Medical Gazette and Journal of Health

**Published:** 1850-10

**Authors:** 


*The New York Medical Gazette and Journal of Health*, is the title of an ably and spiritedly conducted weekly journal, published in the city of New York, under the editorial management of D. Meredith Reese, M. D. It is designed for the non-professional as well as the professional reader, and although it has only been published about five months, we are gratified to learn, that it has already obtained an extensive circulation in nearly all the states of the Union. The editor is unflinching in his opposition to empiricism, and while he is devoting his splendid talents and best energies to the advancement of medical knowledge, he is equally zealous in his efforts to diffuse correct information on all matters relating to the means for the preservation of health among the people at large. Such a paper ought and we trust will be liberally sustained. The dentist would derive as much benefit from its perusal as the physician or general reader.

